# Automated severity scoring of atopic dermatitis patients by a deep neural network

**DOI:** 10.1038/s41598-021-85489-8

**Published:** 2021-03-15

**Authors:** Chul Hwan Bang, Jae Woong Yoon, Jae Yeon Ryu, Jae Heon Chun, Ju Hee Han, Young Bok Lee, Jun Young Lee, Young Min Park, Suk Jun Lee, Ji Hyun Lee

**Affiliations:** 1 Department of Dermatology, Seoul St. Mary’s Hospital, College of Medicine, The Catholic University of Korea, 222, Banpo-daero, Seocho-gu, Seoul, 06591 Korea; 2 Department of Business Management, Kwangwoon University, 536 Nuri Hall, 20, Kwangwoon-ro, Nowon-gu, Seoul, 01897 Korea; 3 Department of Dermatology, Uijeongbu St. Mary’s Hospital, College of Medicine, The Catholic University of Korea, Seoul, Korea

**Keywords:** Epidemiology, Medical research, Risk factors

## Abstract

Scoring atopic dermatitis (AD) severity with the Eczema Area and Severity Index (EASI) in an objective and reproducible manner is challenging. Automated measurement of erythema, papulation, excoriation, and lichenification severity using images has not yet been investigated. Our aim was to determine whether convolutional neural networks (CNNs) could assess erythema, papulation, excoriation, and lichenification severity at a level of competence comparable to dermatologists. We created a standard dataset of 8,000 clinical images showing AD. Each component of the EASI was scored from 0 to 3 by three dermatologists. We trained four CNNs (ResNet V1, ResNet V2, GoogLeNet, and VGG-Net) with the image dataset and determined which CNN was the most suitable for erythema, papulation, excoriation, and lichenification scoring. The brightness of the images in each dataset was adjusted to − 80% to + 80% of the original brightness (i.e., 9 levels by 20%) to investigate if the CNNs accurately measured scores if image brightness levels were changed. Compared to the dermatologists’ scoring, accuracy rates of the CNNs were 99.17% for erythema, 93.17% for papulation, 96.00% for excoriation, and 97.17% for lichenification. CNNs trained with brightness-adjusted images achieved a high accuracy without the need to standardize camera settings. These results suggested that CNNs perform at level of competence comparable to dermatologists for scoring erythema, papulation, excoriation, and lichenification severity.

## Introduction

Atopic dermatitis (AD) is a common skin disease that is characterized by chronic relapsing skin inflammation, disturbed epidermal-barrier function and alterations in various immunological responses including T cells and inflammatory cytokines^1^. Accurate assessment of the extent and severity of AD is essential for quantifying the clinical disease burden and the effectiveness of treatment regimens during testing^2^. For accurate AD severity scoring, more than 15 outcome measurements have been developed. Among these outcome measures for clinical signs of AD, only the Eczema Area and Severity Index (EASI) and the Severity Scoring of Atopic Dermatitis (SCORAD) index show adequate validity^3^. The SCORAD index is validated, but combines subjective assessment of patients’ symptoms with observation of signs^2^. Its disadvantage is that the intraobserver reliability is unclear^3^.

The EASI is valid and internally consistent and has adequate intraobserver reliability, intermediate interobserver reliability, and adequate responsiveness^3^. The EASI also has the strengths of measuring only clinical signs and not patients’ subjective symptoms. Thus, the EASI is used in many clinical studies of AD.

The EASI was designed by modifying the general scheme of the Psoriasis Area and Severity Index (PASI), which is a well-accepted, standardized instrument for assessing therapeutic responses in patients with psoriasis^2^. The EASI consists of four components: erythema, induration/papulation, excoriation, and lichenification, which are scored from 0 to 3 according to severity (none, mild, moderate, and severe). Another important component in measuring the EASI is the affected body surface area, which is divided into head/neck, upper limbs, trunk, and lower limbs, giving 0 to 6 points for the AD-affected area. The EASI score is calculated from the four severity-related components and the affected area points via a mathematical function^2^.

Scoring AD severity with the EASI in an objective and reproducible manner is challenging. To obtain an accurate EASI score, observers must be trained and validated. Therefore, education on EASI scoring is important. However, standardizing conventional educational programs is difficult, as seen for PASI education^4^. In addition, EASI measurements are time consuming and difficult to measure each time a patient visits a clinical setting.

Convolutional neural networks (CNNs) are a branch of deep learning algorithms that have been applied to detect skin cancer, diabetic retinopathy, and onychomycosis^5–8^. In these reports, the accuracy of CNNs trained with a large number of clinical photographs was comparable to specialist clinicians^5–8^. These results were achieved through validation with a large number of clinical photographs and the development of CNNs. Therefore, with a validated dataset of clinical AD photographs, CNNs were expected to be trained to distinguish erythema, induration/induration/papulation, excoriation, and lichenification scores, which are the individual components of the EASI. Our aim was to determine if the CNNs could assess erythema, induration/papulation, excoriation, and lichenification severity at a level of competence comparable to dermatologists. We trained four CNN models (ResNet V1, ResNet V2, GoogLeNet and VGG-Net) with an image dataset and examined which CNN was most suitable for scoring each component of EASI.

## Methods

### Datasets and CNN training

**Figure 1:**
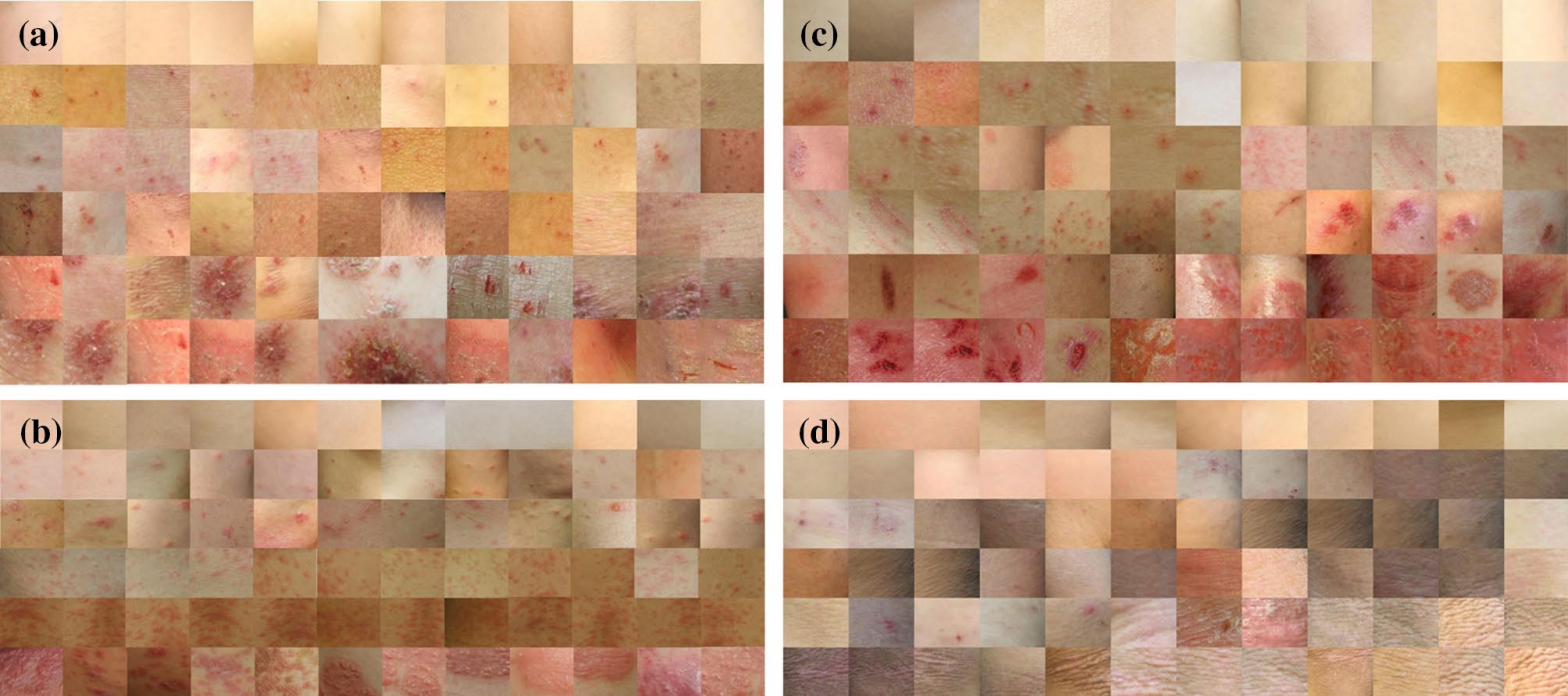
Examples of cropped images of the 4 components of EASI from a standard clinical dataset: (**a**) erythema, (**b**) induration/papulation, (**c**) excoriation and (**d**) lichenification.

**Figure 2:**
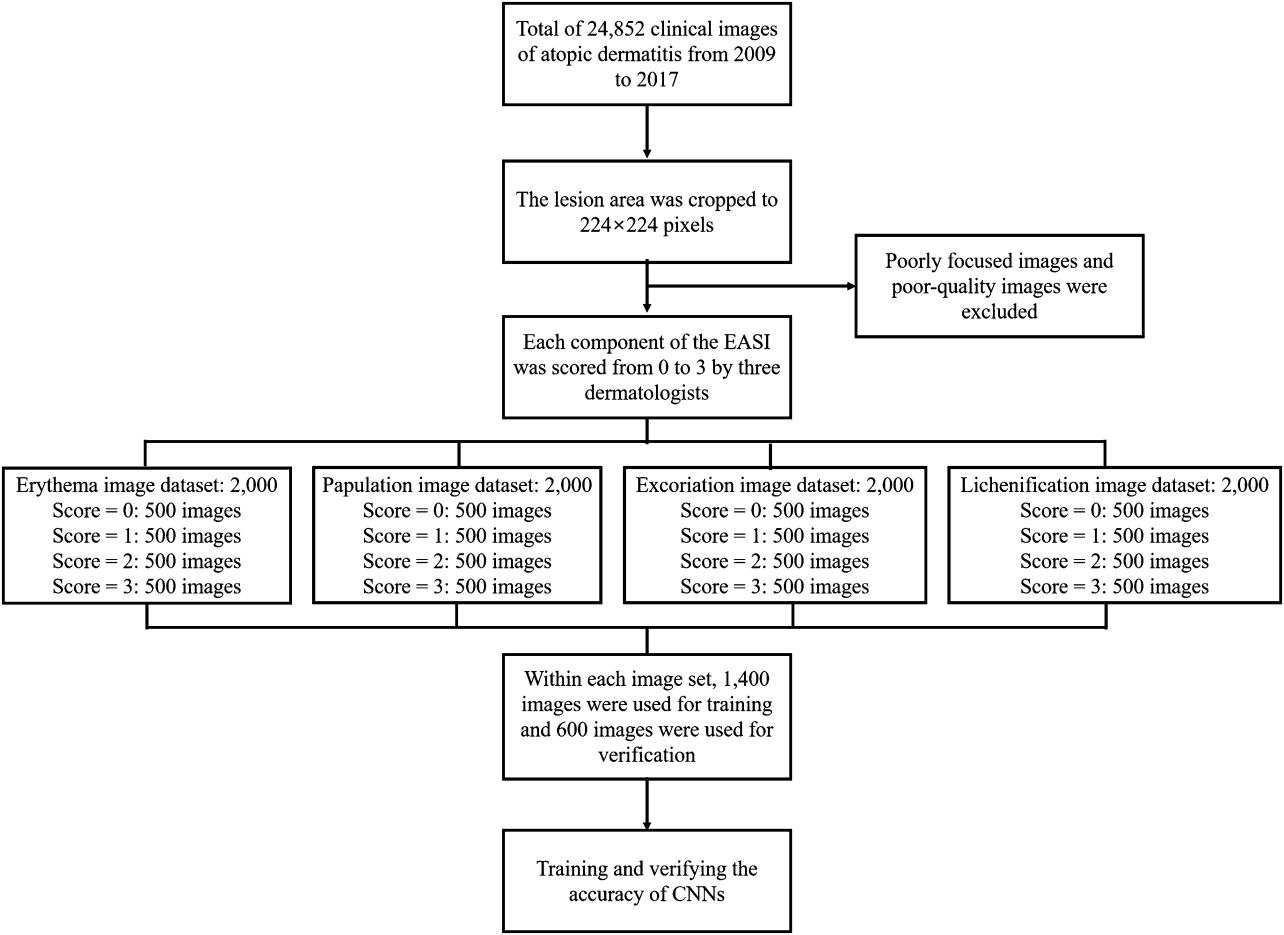
Flow chart of the study.

We used clinical images from Seoul St. Mary’s Hospital to construct AD datasets. Data on the images were collected via a retrospective chart review, and all data were fully anonymized before we accessed them. In total, 24,852 clinical images of AD were acquired from 2009 to 2017, and the lesion area of the images was cropped to 224 by 224 pixels. Poorly focused images and poor-quality images were excluded. Severity of images was scored from 0 to 3 for each component of the EASI by three dermatologists, with the final score determined by consensus among the dermatologists (Fig. 1). For each EASI sign, 500 images were assigned a severity score to create a dataset of 2000 scored images for each EASI component. Of the 8000 cropped images selected, 5600 images (1,400 images each for erythema, induration/papulation, excoriation and lichenification) were used to train the CNNs. The remaining 2400 images (600 images each for erythema, induration/papulation, excoriation and lichenification) were used to validate the CNNs (Fig. 2). For external validation, 400 images each EASI sign were selected from Uijeongbu St. Mary’s hospital in the same way. This study was reviewed and approved by the Institutional Review Board of the Catholic University of Korea (CMC Central IRB: KC18RESI0827).

CNNs such as VGG-Net with 16 and 19 layers (i.e., VGG16 and VGG19); GoogLeNet V1, GoogLeNet V2, GoogLeNet V3, GoogLeNet V4, ResNet V1 with 50, 101, and 152 layers; and ResNet V2 with 50, 101 and 152 layers achieved good performance for image classification in the ImageNet Large Scale Visual Recognition Challenge^9–12^. Additionally, these 12 CNNs achieved excellent performance for dermatology image classification. For this reason, the CNNs were trained in this study to classify the severity of each EASI component.

### Evaluation of the CNNs

The output of the trained CNNs was four continuous numbers between 0 and 1 for each input image that could be interpreted as the probability of each severity level. For example, if an image $\mathrm{X}$ was given to one of CNNs, the output was ${\widehat{y}}_{1}, {\widehat{y}}_{2}, {\widehat{y}}_{3}, \mathrm{and} {\widehat{y}}_{4}$, which were the probabilities of each severity score from 0, 1, 2, and 3, respectively. To identify misclassified severity scores, the specificity and sensitivity of each severity score were analyzed over a change in threshold from 0.01 to 1.00 and their receiver operating characteristic (ROC) curves were plotted using the following equations:

$$Specificity=\frac{number of images with a correctly rejected severity {score}_{N} from the CNN}{number of images that do not belong to severity {score}_{N}}$$

$$Sensitivity=\frac{number of images with a correctly permitted severity {score}_{N} from the CNN}{number of images that belong to severity {score}_{N}}$$

In this study, because trained CNNs performed multiple classification tasks, the performance of each CNN was also analyzed using a confusion matrix. A confusion matrix is a visualization tool typically used in multiclass supervised learning and contains information about the actual classifications and the classifications predicted by a classification model. Each column of the matrix represents the instances in a predicted class, while each row represents the instances in an actual class^9–12^. Furthermore, each element is a number, which is the conditional probability between the predicted outputs obtained from the CNNs and the actual values.

### Adjustment of image brightness

The brightness of the images in each dataset was adjusted to − 80% to + 80% of the original brightness (i.e., 9 levels by 20%) to investigate if the CNNs accurately measured scores if image brightness levels were changed. Additionally, the differences in the accuracy between the CNNs trained with only the original images and those trained with the brightness-adjusted images were investigated.

### Use of human participants

(i) Research was performed in accordance with relevant guidelines/regulations
(ii) Informed consent was obtained from all participants and/or their legal guardians.

## Results

### Accuracy of CNNs compared to dermatologists

The scoring accuracy for erythema, induration/papulation, excoriation, and lichenification according to the CNNs is presented in Table 1. For erythema, ResNet V1 with 101 layers, ResNet V2 with 50 layers and ResNet V2 with 152 layers each achieved a high accuracy of 99.17%. The accuracies of ResNet V1 with 50 layers, ResNet V1 with 152 layers, ResNet V2 with 101 layers were 99.00%, 98.83%, and 98.83%, respectively. While each ResNet model achieved an accuracy greater than 98%, the maximum accuracy achieved by GoogLeNet was 96.67% (GoogLeNet V2). The accuracy levels achieved by VGG16 and VGG19 were 95.67% and 94.33%.

**Table 1:** Scoring accuracy of erythema, induration/papulation, excoriation, and lichenification according to the CNN algorithms.

CNN algorithm		Erythema (%)	Induration/population (%)	Excoriation (%)	Lichenification (%)
GoogLeNet	V1	95.00	82.00	86.50	85.50
	V2	96.67	86.17	93.00	83.50
	V3	92.67	85.00	91.17	82.50
	V4	93.50	76.83	84.50	80.67
ResNet V1	50	99.00	93.17	94.50	97.00
	101	99.17	91.67	94.67	97.17
	152	98.83	77.33	92.83	97.00
ResNet V2	50	99.17	83.50	96.00	91.00
	101	98.83	88.33	85.83	92.33
	152	99.17	73.00	85.83	89.00
VGG-Net	16	95.67	90.17	94.00	94.50
	19	94.33	90.83	91.33	92.83

For induration/papulation, ResNet V1 with 50 layers achieved a 93.17% accuracy compared to the dermatologists. The accuracy of ResNet V1 with 101 layers was 91.67%, and the accuracies of VGG19 and VGG16 were 90.83%, and 90.17%. GoogLeNet achieved a relatively low accuracy (less than 86.17%) compared to other CNNs.

For the excoriation measurement, ResNet V2 with 50 layers achieved the highest accuracy of 96.00%. ResNet V1 with 101 layers and ResNet V1 with 50 layers achieved, respectively, 94.67% and 94.50% accuracy. The accuracies of VGG16 and VGG19 were 93.00% and 91.33%. GoogLeNet V2 achieved accuracy 93.00% and V3 achieved 91.17%, with other versions achieving less than 90% accuracy.

Lichenification had relatively low accuracy levels compared to other components. ResNet V1 with 101 layers achieved an accuracy of 97.17%. The accuracy of ResNet V1 was 97.00% for both 50 and 152 layers. The other CNN algorithms achieved less than 94.5% accuracy.

**Figure 3:**
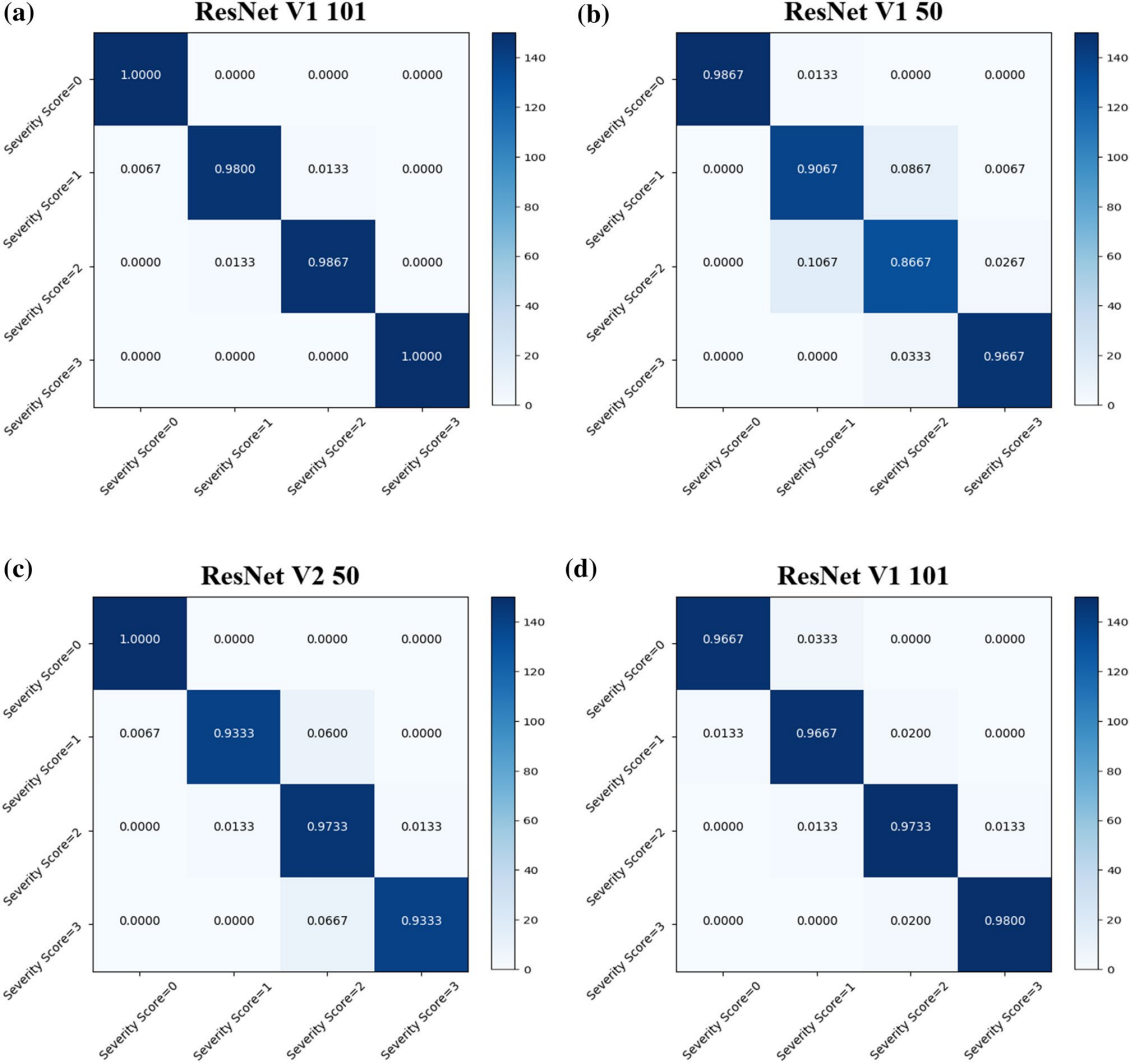
The most accurate CNN confusion matrices for (**a**) erythema, (**b**) induration/papulation, (**c**) excoriation and (**d**) lichenification.

Confusion matrices for erythema, induration/papulation, excoriation, and lichenification for the CNNs are in Fig. 3. The misclassification probabilities of the CNNs occurred mainly between severity scores of 1 and 2, but the probabilities were not high.

As a result of verifying our model with the Uijeongbu St. Mary’s hospital dataset, the accuracy of severity scoring was 90.63% for erythema, 89.06% for induration/papulation, 87.50% for excoriation and 85.94% for lichenification.

### Accuracy changes according to brightness

**Figure 4:**
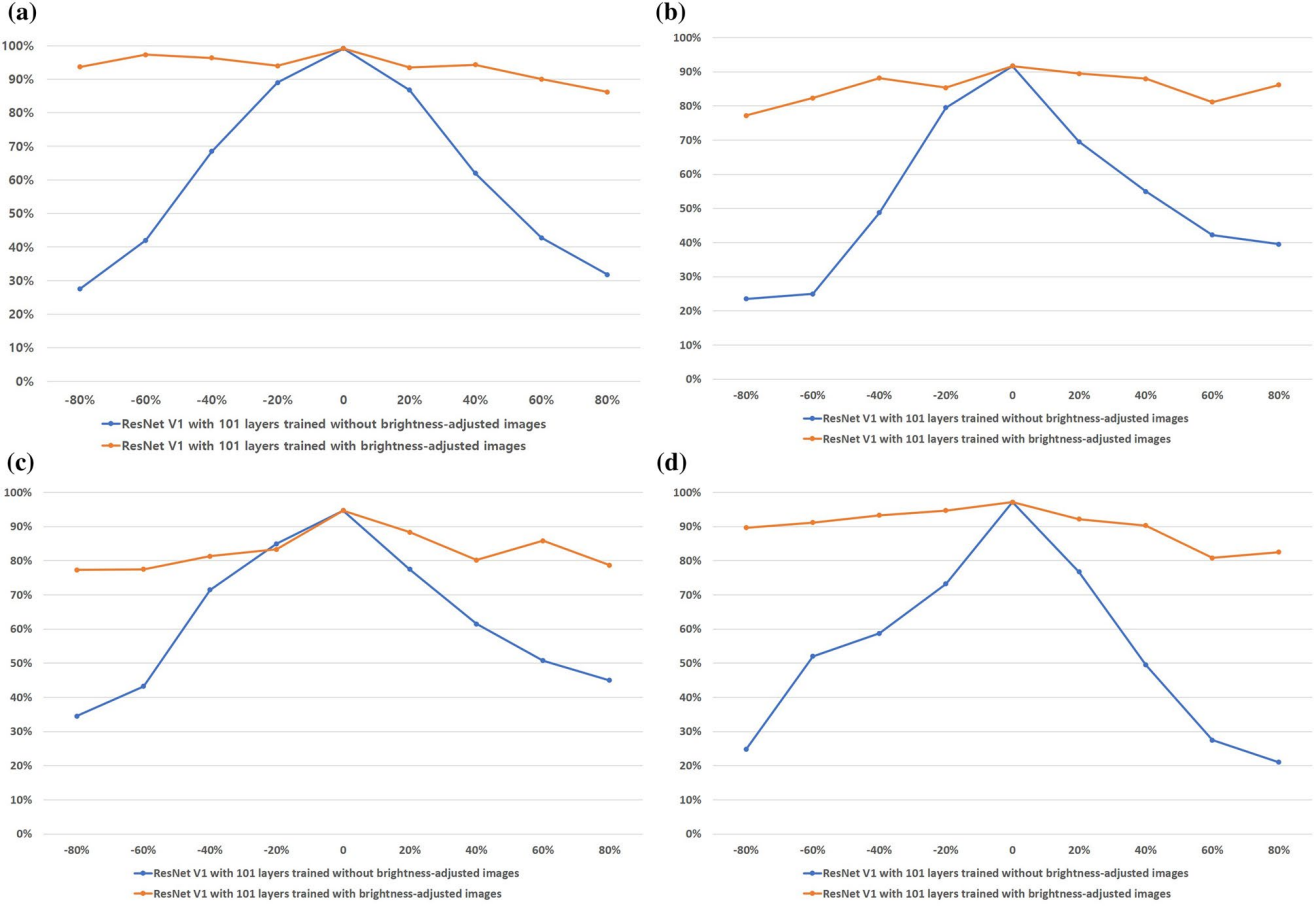
Comparison of ResNet V1 101 trained with and without brightness-adjusted images: (**a**) erythema, (**b**) induration/papulation, (**c**) excoriation and (**d**) lichenification.

CNNs trained with the original images showed that severity scoring accuracy decreased as the degree of brightness adjustment increased. For ResNet V1 with 101 layers, adjusting the brightness of the image dataset by 0%, − 20%, − 40%, − 60%, and − 80%, respectively reduced the accuracy of erythema scores from 98.9% to 89.0%, 68.5%, 42.0%, and 27.5% (Fig. 4a). Adjusting the brightness of the image dataset by 0%, + 20%, + 40%, + 60%, and + 80% respectively reduced the accuracy of erythema scores from 98.9% to 86.6%, 62.0%, 42.6%, and 31.8%. When CNNs were trained with all brightness-adjusted images, the accuracy of ResNet V1 with 101 layer improved to over 86% (Fig. 4a). These results were similar for induration/papulation, excoriation, and lichenification scores (Fig. 4b–d), and other CNNs yielded similar results (Supplemental Tables S1 and S2).

## Discussion

This paper used CNNs to measure AD severity. The application of deep neural networks in dermatology is mainly limited to the diagnosis of skin cancers^5,13–17^. Although making diagnoses through a deep neural network is important, replacing time-consuming tasks for physicians through a deep neural network is also important. One of these tasks is measuring the EASI score in dermatology. The use of CNNs may increase the accuracy of AD severity scoring, allowing an accurate treatment response for patients and, improving rapport with patients to improve treatment compliance.

The EASI is an investigator-assessed instrument identified as one of the three best-validated outcome measures for AD^18,19^. The EASI was chosen by the International Harmonizing Outcomes Measures for Eczema initiative, after extensive systematic evaluation of its measurement properties, as the preferred core instrument to measure clinical signs in all future AD trials^19,20^. Currently, the EASI is used often in clinical practice and trials of AD.

The problems with measuring the EASI are that it is time consuming and has intermediate interobserver reliability. Training in the EASI takes approximately 30 min^2,21^. The time required to measure the EASI in one patient is 6.0 ± 4.5 min (mean ± SD)^19^. EASI training does not take much time, but checking the EASI in complicated cases can take as long as 10 min, reducing the time for patient care and education in clinical practice settings. Improving interobserver reliability requires validation between observers, which increases the training time for EASI and requires educational lectures and reference photographs^4^. Therefore, a reliable measuring system could support observers, improve interobserver reliability and shorten measuring time of EASI. Studies are underway to develop a reliable measuring system for diseases such as melasma, vitiligo, and psoriasis^22–24^. For AD, a deep neural network may solve these problems. Deep neural networks, including CNNs, achieve state-of-the-art performance in numerous vision tasks, including image classification, object detection, and segmentation. However, no reports have applied CNNs to measure severity scores in skin diseases, including AD.

According to our results, for erythema and lichenification scoring, ResNet V1 with 101 layers achieved an accuracy greater than 99%. Erythema is a component confirmed by degree of redness, and seems to allow high accuracy because few factors affected the CNNs. The lichenification score is determined by skin thickness and wrinkle depth. In clinical photographs, depth of wrinkles tends to be represented by shadows that are relatively dark compared to the surrounding skin. Since this tendency is clear for lichenification, CNNs may have shown the high accuracy of 97%. However, since recognizing the depth in a 2-dimensional image is difficult and induration/papulation is often accompanied by erythema, those severity scores may be less accurate (e.g., 93% for of induration/papulation). As CNNs become more accurate and as the amount of training data increases, we expect that training with more data will overcome these limitations.

External validation results with the Uijeongbu St. Mary’s Hospital dataset showed that the accuracy of our model was 85% to 90% for each component of EASI. These results appeared to be due to the intermediate interobserver reliability of EASI. If dermatologists from Uijeongbu and Seoul St. Mary’s hospital scored the severity of each component of EASI in agreement, the results might also have high accuracy. This result means that more accurate models could be created if more dermatologists participated, and it is expected to create models that can be used globally in the future.

Standardizing camera conditions such as the shutter speed, iris, and film speed are thought to be necessary to standardize the light intensity or brightness of photographs when taking clinical images in dermatology clinics^6^. Since not all clinical images can be taken under the same conditions in the real world, the brightness of clinical pictures was adjusted and used to train the CNNs. The result was a large difference in the accuracy of the severity scoring between CNNs trained with the brightness-adjusted images and CNNs not trained with the brightness-adjusted images. Training with the brightness-adjusted images was also effective at inflating the size of the dataset, which seemed to increase the accuracy. This process can be automated through the program and is recommended to increase the accuracy of CNNs.

This system had some limitations. The system would be better if more clinical images per EASI component had been used to train the CNNs. This study was conducted on Korean population, and the Fitzpatrick skin type of Koreans is usually 2–4. Therefore, darker skin patients were not included in this study. However, we suspect that this method could also work in a dark skin population with appropriate adjustments. This study was a pilot to investigate if CNNs could be used for EASI scoring and the CNNs achieved a high accuracy. In order to measure the EASI, the severity score of each component and the ratio of the lesion area is required, and further study is needed to determine how to recognize the area score automatically.

The results from this pilot study suggest that CNNs could be used for clinical scoring of atopic dermatitis and to assist dermatologists in measuring the EASI.

## Supplementary Information

Supplementary Information.

## Abbreviations

AD: Atopic dermatitis
CNNs: Convolutional neural networks
EASI: Eczema Area and Severity Index
PASI: Psoriasis Area and Severity Index
SCORAD: Severity Scoring of Atopic Dermatitis
