# Author Correction: Automated severity scoring of atopic dermatitis patients by a deep neural network

**DOI:** 10.1038/s41598-021-94433-9

**Published:** 2021-07-27

**Authors:** Chul Hwan Bang, Jae Woong Yoon, Jae Yeon Ryu, Jae Heon Chun, Ju Hee Han, Young Bok Lee, Jun Young Lee, Young Min Park, Suk Jun Lee, Ji Hyun Lee

**Affiliations:** 1 Department of Dermatology, Seoul St. Mary’s Hospital, College of Medicine, The Catholic University of Korea, 222, Banpo-daero, Seocho-gu, Seoul, 06591 Korea; 2 Department of Business Management, Kwangwoon University, 536 Nuri Hall, 20, Kwangwoon-ro, Nowon-gu, Seoul, 01897 Korea; 3 Department of Dermatology, Uijeongbu St. Mary’s Hospital, College of Medicine, The Catholic University of Korea, Seoul, Korea

Correction to: *Scientific Reports* 10.1038/s41598-021-85489-8, published online 15 March 2021

The Acknowledgements section in the original version of this Article was incomplete.

“This study was supported by the National Research Foundation of Korea (NRF) grant funded by the Korea government (2018R1D1A1B07044100) and the Institute of Information & Communications, Technology Planning & Evaluation (IITP) grant funded by the Korean government (2017-0-00855).”

now reads:

“This study was supported by the National Research Foundation of Korea (NRF) grant funded by the Korea government (2018R1D1A1B07044100) and the Institute of Information & Communications, Technology Planning & Evaluation (IITP) grant funded by the Korean government (2017-0-00855). This work was supported by the Research Grant of Kwangwoon University in 2020."

The original Article has been corrected.

