# Supplementary material for: Automated severity scoring of atopic dermatitis patients by a deep neural network
Source: Sci Rep. 2021 Mar 15;11:6049. doi: 10.1038/s41598-021-85489-8 (PMC7961024; doi:10.1038/s41598-021-85489-8)
Supplement: Supplementary file 1 — Supplementary Information. [file 41598_2021_85489_MOESM1_ESM.docx]

**Article type:** Original article

**Title: Automated severity scoring of atopic dermatitis patients by a deep neural network**

Chul Hwan Bang, MD;^1#^ Jae Woong Yoon, MS;^2#^ Jae Yeon Ryu, MD;^1^ Jae Heon Chun, MS;^2^ Ju Hee Han, MD;^1^ Young Bok Lee, MD, PhD;^3^ Jun Young Lee, MD, PhD;^1^ Young Min Park, MD, PhD;^1^ Suk Jun Lee, PhD;^2*^ and Ji Hyun Lee, MD, PhD^1*^

^1^Department of Dermatology, Seoul St. Mary’s Hospital, College of Medicine, The Catholic University of Korea, Seoul, Korea

^2^Department of Business Management, Kwangwoon University, Seoul, Korea

^3^Department of Dermatology, Uijeongbu St. Mary’s Hospital, College of Medicine, The Catholic University of Korea, Seoul, Korea

***Correspondence and reprint requests should be addressed to:**

Ji Hyun Lee, MD, PhD

Department of Dermatology, Seoul St. Mary’s Hospital, College of Medicine, The Catholic University of Korea, 222, Banpo-daero, Seocho-gu, Seoul, 06591, Republic of Korea

Tel.: + 82-2-2258-1395

Fax: + 82-2-599-9950

E-mail: ejee@catholic.ac.kr

Suk Jun Lee, PhD

Business School, Kwangwoon University, 536 Nuri Hall, 20, Kwangwoon-ro, Nowon-gu, Seoul, 01897, Republic of Korea

Tel.: + 82-2-940-8103

Fax: + 82-2-940-8181

E-mail: sjlee@kw.ac.kr

^#^Chul Hwan Bang and Jae Woong Yoon contributed equally to this work as co-first authors.

*Ji Hyun Lee and Suk Jun Lee contributed equally to this work as the corresponding authors.

Supplemental Table 1. Accuracy of the EASI components with image-brightness correction in CNNs not trained with brightness-adjusted images

|  | (%) | GoogLeNetV1 (%) | GoogLeNetV2 (%) | GoogLeNetV3 (%) | GoogLeNetV4 (%) | ResNet_V1_50 (%) | ResNet_V1_101 (%) | ResNet_V1_152 (%) | ResNet_V2_50 (%) | ResNet_V2_101 (%) | ResNet_V2_152 (%) | VGG_16 (%) | VGG_19 (%) |
| --- | --- | --- | --- | --- | --- | --- | --- | --- | --- | --- | --- | --- | --- |
| Erythema | -80 | 33.75 | 27.50 | 31.75 | 34.25 | 31.50 | 27.50 | 35.25 | 25.00 | 25.00 | 25.00 | 31.25 | 44.25 |
|  | -60 | 25.00 | 47.75 | 47.75 | 58.50 | 40.75 | 42.00 | 54.25 | 25.25 | 26.75 | 29.25 | 72.50 | 72.00 |
|  | -40 | 28.50 | 75.50 | 80.75 | 76.75 | 63.00 | 68.50 | 68.75 | 59.00 | 65.00 | 60.75 | 82.50 | 85.75 |
|  | -20 | 37.00 | 95.50 | 91.00 | 88.25 | 88.00 | 89.00 | 88.50 | 87.75 | 87.50 | 86.25 | 90.75 | 91.25 |
|  | **0** | **95.00** | **96.67** | **92.67** | **93.50** | **99.00** | **99.17** | **98.83** | **99.17** | **98.83** | **99.17** | **95.67** | **94.33** |
|  | +20 | 55.50 | 90.50 | 90.75 | 78.75 | 89.25 | 86.75 | 94.00 | 83.75 | 84.50 | 93.25 | 93.50 | 94.25 |
|  | +40 | 55.75 | 82.00 | 80.50 | 60.50 | 76.75 | 62.00 | 79.50 | 69.25 | 65.00 | 79.50 | 88.25 | 87.25 |
|  | +60 | 57.00 | 72.50 | 69.50 | 43.50 | 63.00 | 42.75 | 64.00 | 65.25 | 52.25 | 64.50 | 80.75 | 79.25 |
|  | +80 | 51.50 | 64.75 | 60.00 | 33.25 | 56.50 | 31.75 | 51.00 | 55.50 | 46.25 | 54.75 | 72.50 | 71.00 |
| Induration / Papulation | -80 | 18.25 | 27.75 | 24.75 | 33.50 | 24.75 | 23.50 | 26.50 | 27.75 | 25.50 | 24.75 | 31.50 | 31.00 |
|  | -60 | 35.50 | 45.50 | 47.25 | 31.00 | 39.00 | 25.00 | 25.75 | 28.75 | 19.25 | 29.75 | 50.50 | 51.00 |
|  | -40 | 56.25 | 49.25 | 65.75 | 56.00 | 58.75 | 48.75 | 53.25 | 50.00 | 48.25 | 56.50 | 62.00 | 75.75 |
|  | -20 | 68.75 | 56.50 | 71.75 | 69.00 | 79.50 | 79.50 | 65.75 | 71.75 | 69.50 | 66.00 | 76.25 | 80.75 |
|  | **0** | **82.00** | **86.17** | **85.00** | **76.83** | **93.17** | **91.67** | **77.33** | **83.50** | **88.33** | **73.00** | **90.17** | **90.83** |
|  | +20 | 70.00 | 70.25 | 69.50 | 59.50 | 73.25 | 69.50 | 70.75 | 76.00 | 72.00 | 57.25 | 83.50 | 84.00 |
|  | +40 | 67.25 | 66.50 | 59.00 | 47.00 | 55.50 | 55.00 | 52.75 | 67.50 | 59.50 | 40.25 | 81.75 | 81.50 |
|  | +60 | 71.25 | 62.75 | 51.00 | 42.75 | 44.75 | 42.25 | 40.00 | 50.00 | 49.25 | 34.50 | 77.75 | 76.25 |
|  | +80 | 66.00 | 59.00 | 44.25 | 37.75 | 38.00 | 39.50 | 32.75 | 46.25 | 40.00 | 31.25 | 71.75 | 67.75 |
| Excoriation | -80 | 24.75 | 29.00 | 44.75 | 28.00 | 23.00 | 34.50 | 28.25 | 25.00 | 25.25 | 25.00 | 33.75 | 25.00 |
|  | -60 | 57.25 | 56.75 | 56.75 | 63.00 | 37.25 | 43.25 | 50.00 | 31.50 | 27.75 | 35.75 | 51.00 | 62.00 |
|  | -40 | 80.75 | 82.75 | 76.50 | 76.00 | 57.25 | 71.50 | 75.25 | 47.50 | 59.50 | 65.50 | 67.50 | 71.75 |
|  | -20 | 83.50 | 89.54 | 88.25 | 82.50 | 77.50 | 85.00 | 85.25 | 77.75 | 79.50 | 83.75 | 87.00 | 86.50 |
|  | **0** | **86.50** | **93.00** | **91.17** | **84.50** | **94.50** | **94.67** | **92.83** | **96.00** | **85.83** | **85.83** | **94.00** | **91.33** |
|  | +20 | 81.00 | 89.25 | 90.75 | 80.50 | 76.00 | 77.50 | 77.00 | 75.25 | 79.25 | 82.00 | 79.25 | 83.25 |
|  | +40 | 76.00 | 85.00 | 86.00 | 72.25 | 63.50 | 61.50 | 56.75 | 57.00 | 79.50 | 81.75 | 78.75 | 82.00 |
|  | +60 | 70.00 | 80.25 | 79.00 | 61.50 | 57.00 | 50.75 | 46.75 | 50.50 | 71.00 | 72.50 | 76.25 | 80.75 |
|  | +80 | 68.00 | 74.00 | 73.50 | 52.00 | 54.75 | 45.00 | 38.25 | 45.50 | 64.25 | 68.00 | 75.50 | 75.25 |
| Lichenification | -80 | 34.75 | 23.50 | 33.25 | 27.75 | 13.25 | 24.75 | 26.50 | 26.50 | 24.50 | 25.00 | 27.25 | 42.75 |
|  | -60 | 55.00 | 48.25 | 44.00 | 43.00 | 46.50 | 52.00 | 50.00 | 41.75 | 37.50 | 24.75 | 42.00 | 51.50 |
|  | -40 | 67.75 | 55.00 | 53.25 | 60.00 | 59.00 | 58.75 | 66.50 | 53.00 | 53.75 | 59.72 | 53.75 | 63.50 |
|  | -20 | 73.00 | 62.25 | 73.50 | 64.50 | 75.00 | 73.25 | 76.75 | 77.25 | 68.25 | 76.50 | 77.25 | 80.25 |
|  | **0** | **85.50** | **83.50** | **82.50** | **80.67** | **97.00** | **97.17** | **97.00** | **91.00** | **92.33** | **89.00** | **94.50** | **92.83** |
|  | +20 | 79.50 | 70.00 | 79.75 | 71.50 | 83.25 | 76.75 | 81.75 | 76.25 | 87.50 | 77.50 | 80.00 | 78.75 |
|  | +40 | 75.25 | 66.25 | 75.00 | 53.75 | 65.75 | 49.50 | 64.50 | 66.00 | 79.50 | 76.00 | 69.75 | 70.25 |
|  | +60 | 66.00 | 65.50 | 67.75 | 42.50 | 42.75 | 27.50 | 47.50 | 57.00 | 63.25 | 61.50 | 60.50 | 57.50 |
|  | +80 | 60.25 | 62.75 | 59.25 | 35.75 | 30.75 | 21.00 | 31.50 | 50.50 | 52.75 | 45.50 | 51.50 | 50.50 |

Supplemental Table 2. Accuracy of the EASI components with image-brightness correction in CNNs trained with brightness-adjusted images

|  | (%) | GoogLeNetV1 (%) | GoogLeNetV2 (%) | GoogLeNetV3 (%) | GoogLeNetV4 (%) | ResNet_V1_50 (%) | ResNet_V1_101 (%) | ResNet_V1_152 (%) | ResNet_V2_50 (%) | ResNet_V2_101 (%) | ResNet_V2_152 (%) | VGG_16 (%) | VGG_19 (%) |
| --- | --- | --- | --- | --- | --- | --- | --- | --- | --- | --- | --- | --- | --- |
| Erythema | -80 | 75.33 | 52.50 | 66.50 | 65.50 | 92.83 | 93.67 | 85.83 | 87.17 | 79.00 | 72.83 | 58.17 | 51.50 |
|  | -60 | 83.83 | 65.17 | 74.50 | 72.50 | 91.67 | 97.33 | 85.17 | 84.00 | 92.83 | 77.83 | 92.83 | 93.17 |
|  | -40 | 87.00 | 56.33 | 81.67 | 71.67 | 91.00 | 96.33 | 87.67 | 89.50 | 88.17 | 80.50 | 97.50 | 97.67 |
|  | -20 | 83.17 | 49.00 | 82.50 | 73.50 | 95.17 | 94.00 | 88.67 | 87.67 | 90.50 | 83.00 | 96.50 | 96.83 |
|  | **0** | **95.00** | **96.67** | **92.67** | **93.50** | **99.00** | **99.17** | **98.83** | **99.17** | **98.83** | **99.17** | **95.67** | **94.33** |
|  | +20 | 85.67 | 74.00 | 81.33 | 74.50 | 95.33 | 93.50 | 82.50 | 88.50 | 89.33 | 57.17 | 95.67 | 94.67 |
|  | +40 | 81.50 | 74.50 | 78.50 | 78.83 | 91.83 | 94.33 | 78.00 | 87.00 | 89.17 | 77.67 | 93.17 | 93.17 |
|  | +60 | 78.33 | 71.17 | 74.67 | 62.83 | 89.00 | 90.00 | 64.17 | 79.17 | 85.50 | 74.17 | 90.83 | 91.33 |
|  | +80 | 63.00 | 70.17 | 71.17 | 57.33 | 86.17 | 86.17 | 50.83 | 71.50 | 82.50 | 76.33 | 86.83 | 87.17 |
| Induration / Papulation | -80 | 56.67 | 51.33 | 60.83 | 60.17 | 80.17 | 77.17 | 74.00 | 76.33 | 75.33 | 67.00 | 52.83 | 77.50 |
|  | -60 | 70.67 | 67.50 | 73.00 | 70.00 | 82.50 | 82.33 | 70.67 | 80.50 | 81.83 | 66.33 | 83.17 | 82.00 |
|  | -40 | 74.50 | 63.00 | 73.50 | 68.83 | 82.00 | 88.17 | 72.17 | 78.00 | 84.00 | 70.17 | 87.00 | 81.00 |
|  | -20 | 76.17 | 70.00 | 75.83 | 63.33 | 81.17 | 85.33 | 74.83 | 83.50 | 81.50 | 69.67 | 80.50 | 87.50 |
|  | **0** | **82.00** | **86.17** | **85.00** | **76.83** | **93.17** | **91.67** | **77.33** | **83.50** | **88.33** | **73.00** | **90.17** | **90.83** |
|  | +20 | 75.17 | 75.17 | 71.50 | 66.67 | 82.33 | 89.50 | 71.50 | 78.83 | 80.00 | 72.17 | 84.83 | 83.67 |
|  | +40 | 74.00 | 72.67 | 67.33 | 70.00 | 83.67 | 88.00 | 68.33 | 85.83 | 79.67 | 64.67 | 88.50 | 84.83 |
|  | +60 | 70.83 | 72.33 | 68.50 | 61.50 | 86.33 | 81.17 | 61.50 | 76.50 | 78.00 | 71.83 | 90.17 | 89.50 |
|  | +80 | 66.50 | 69.50 | 67.67 | 56.67 | 88.17 | 86.17 | 54.33 | 79.83 | 78.33 | 69.67 | 83.67 | 87.00 |
| Excoriation | -80 | 67.50 | 64.33 | 60.83 | 62.00 | 76.83 | 77.33 | 63.83 | 71.50 | 81.83 | 65.50 | 74.83 | 67.67 |
|  | -60 | 78.83 | 70.50 | 74.33 | 71.17 | 77.33 | 77.50 | 72.33 | 81.17 | 82.50 | 66.00 | 85.67 | 82.50 |
|  | -40 | 72.83 | 67.50 | 77.50 | 72.50 | 76.67 | 81.33 | 69.00 | 78.50 | 79.50 | 66.50 | 88.67 | 87.33 |
|  | -20 | 73.33 | 61.33 | 79.17 | 71.50 | 84.00 | 83.33 | 69.17 | 82.50 | 84.17 | 66.83 | 87.17 | 88.67 |
|  | **0** | **86.50** | **93.00** | **91.17** | **84.50** | **94.50** | **94.67** | **92.83** | **96.00** | **85.83** | **85.83** | **94.00** | **91.33** |
|  | +20 | 76.33 | 67.33 | 72.33 | 71.00 | 80.17 | 88.33 | 72.17 | 78.33 | 83.33 | 66.33 | 89.50 | 88.00 |
|  | +40 | 79.33 | 73.50 | 76.17 | 71.00 | 82.17 | 80.17 | 75.17 | 79.00 | 81.17 | 69.50 | 86.67 | 90.83 |
|  | +60 | 72.67 | 76.50 | 72.50 | 65.83 | 79.17 | 85.83 | 62.67 | 75.50 | 82.67 | 68.33 | 86.00 | 88.67 |
|  | +80 | 73.33 | 74.00 | 72.83 | 56.83 | 80.33 | 78.67 | 74.00 | 77.67 | 77.83 | 69.67 | 84.83 | 86.83 |
| Lichenification | -80 | 59.50 | 54.00 | 69.17 | 63.67 | 87.00 | 89.67 | 76.17 | 83.17 | 83.67 | 68.83 | 81.67 | 81.83 |
|  | -60 | 73.50 | 66.17 | 72.83 | 69.50 | 90.33 | 91.17 | 78.83 | 87.50 | 85.17 | 73.00 | 87.83 | 88.33 |
|  | -40 | 77.50 | 69.83 | 77.17 | 73.33 | 91.67 | 93.33 | 80.00 | 88.67 | 87.00 | 72.50 | 92.33 | 91.50 |
|  | -20 | 79.67 | 63.33 | 77.67 | 72.00 | 94.17 | 94.67 | 76.00 | 84.17 | 89.33 | 70.67 | 91.50 | 91.17 |
|  | **0** | **85.50** | **83.50** | **82.50** | **80.67** | **97.00** | **97.17** | **97.00** | **91.00** | **92.33** | **89.00** | **94.50** | **92.83** |
|  | +20 | 79.67 | 75.00 | 76.83 | 70.50 | 93.83 | 92.17 | 77.00 | 84.33 | 90.33 | 68.00 | 89.00 | 91.67 |
|  | +40 | 79.00 | 75.50 | 79.67 | 75.50 | 89.67 | 90.33 | 74.00 | 82.17 | 86.67 | 71.83 | 89.17 | 89.83 |
|  | +60 | 73.83 | 73.00 | 82.67 | 74.00 | 88.33 | 80.83 | 69.33 | 78.50 | 80.00 | 68.67 | 86.67 | 87.33 |
|  | +80 | 72.83 | 73.83 | 77.67 | 66.00 | 84.83 | 82.50 | 70.33 | 81.00 | 78.50 | 64.67 | 79.50 | 72.50 |
